# Impact of geniohyoid and masseter muscle masses on dysphagia after salvage surgery and radiotherapy in head and neck cancer

**DOI:** 10.1038/s41598-021-82039-0

**Published:** 2021-01-26

**Authors:** Nao Hashida, Hiroshi Shamoto, Keisuke Maeda, Hidetaka Wakabayashi

**Affiliations:** 1 Department of Rehabilitation, Osaka International Cancer Institute, Osaka City, Japan; 2 Takano Hospital, Futaba-County, Fukushima, Japan; 3 Department of Disaster and Comprehensive Medicine, Fukushima Medical University, Fukushima City, Fukushima Japan; 4 Department of Geriatric Medicine, National Center for Geriatrics and Gerontology, 7-430 Morioka, Obu, Aichi 474-8511 Japan; 5 Department of Rehabilitation Medicine, Tokyo Women’s Medical University Hospital, Tokyo, Japan

**Keywords:** Signs and symptoms, Head and neck cancer

## Abstract

This study aimed to determine whether geniohyoid and/or masseter muscle mass can predict the severity of dysphagia after salvage surgery for head and neck cancer. We conducted a retrospective cohort study of 45 male patients with head and neck cancer (median age, 68 years) who underwent salvage surgery. The preoperative geniohyoid and masseter muscle masses were evaluated using computed tomography and the severity of dysphagia was evaluated by Penetration Aspiration Scale (PAS), Functional Oral Intake Scale (FOIS) and Oropharyngeal swallow efficiency (OPSE). The median PAS, FOIS and OPSE scores after surgery were 7 (interquartile range [IQR] 1–8), 6 (IQR 2–7) and 95.8 (IQR 67.1–116.2), respectively. The mean geniohyoid muscle masses were 3.13 ± 0.78 cm^2^ and the mean masseter muscle masses were 4.37 ± 0.99 cm^2^, respectively. The multivariate analysis showed that the geniohyoid muscle mass was significantly associated with the PAS, FOIS and OPSE scores. Conversely, the masseter muscle mass was not significantly associated with the PAS score but was significantly associated with the FOIS and OPSE scores. Geniohyoid muscle mass may predict the severity of dysphagia after salvage surgery.

## Introduction

Dysphagia is a common complication associated with head and neck cancer treatment and may lead to aspiration pneumonia, choking, dehydration, malnutrition, lower quality of life, and/or premature death^1–4^. Patients who undergo salvage surgery after radiotherapy are at a higher risk of developing severe dysphagia compared to that before initial treatment^5,6^ and at high risk of developing malnutrition and muscle wasting owing to pain in the oral and pharyngeal mucous membranes, dry mouth, dysgeusia, and dysphagia due to radiotherapy^7–9^. Salvage surgery without total laryngectomy may lead to good oncologic outcomes but is associated with unpredictable functional deficits affecting the voice and swallowing ability, high complication rates of aspiration pneumonia, and increased risk of prolonged hospitalization^6,10–12^. Furthermore, there is controversy regrading this procedure with respect to the swallowing function, and patients occasionally experience severe dysphagia^12–14^. In such cases, total laryngectomy may be required to enable patients to eat without aspiration.

Decreased skeletal muscle mass is associated with complications after cancer surgery^15^. Loss of whole-body skeletal muscle mass followed by loss of swallowing muscle mass and function leads to sarcopenic dysphagia^16–18^. Furthermore, sarcopenia is a risk factor for dysphagia in elderly patients^19^. In patients with cancer, the skeletal muscle mass is associated with the swallowing function^20^; therefore, we hypothesized that skeletal muscle mass prior to surgery may predict the swallowing function after salvage surgery for head and neck cancer. Recently, the masseter muscle mass was used to assess the skeletal muscle mass^21^. A previous electromyographic study has reported that the masseter muscle is the strongest muscle among the masticatory muscles with respect to chewing hard food^22^. In addition, inadequate oral intake and inappropriate food texture affect disuse atrophy and weakness of the masseter muscle^22^. Thus, we hypothesized that the masseter muscle is associated with oropharyngeal dysphagia due to sarcopenia. The geniohyoid muscle is involved in swallowing, and two of the suprahyoid muscles play important roles in the pharyngeal phase of swallowing; furthermore, reduced geniohyoid muscle mass may be associated with dysphagia^23,24^. In addition, the geniohyoid muscle is the main muscle, among the suprahyoid muscles, involved in hyoid bone movement^25^. The geniohyoid muscle was also strongly associated with severe dysphagia compared with other suprahyoid muscles when it is in the radiation field^26^.

Therefore, we visualized the preoperative geniohyoid and masseter muscle masses, which were related to dysphagia or sarcopenia, via neck computed tomography (CT) used for cancer status evaluation and determined whether these muscle masses may predict the severity of dysphagia after salvage surgery in patients who had previously undergone radiotherapy for head and neck cancer.

## Methods

### Patients

We conducted a retrospective cohort study with male patients who underwent salvage surgery for recurrent/remnant tumors or necrosis of tumors after radiotherapy for head and neck cancer in the Department of Otolaryngology—Head and Neck Surgery at Osaka Medical Center for Cancer and Cardiovascular Diseases (present facility name: Osaka International Cancer Institute) and had been referred to the Department of Rehabilitation for dysphagia rehabilitation between 2012 and 2016. We excluded patients who underwent surgeries that were less invasive and did not cause dysphagia and had not been referred for rehabilitation.

Patients who had undergone total laryngectomy and resection of the geniohyoid muscle or ansa cervicalis as well as those who had not undergone neck CT before surgery were also excluded. Moreover, patients with oral cancer were excluded because the geniohyoid muscles were resected in most cases. Postoperatively, all patients participated in a dysphagia rehabilitation program five times a week, coordinated by a speech-language-hearing therapist (ST) during hospitalization. Dysphagia rehabilitation involved dysphagia assessment, range of motion exercises, resistance exercises, and swallowing training using compensatory techniques. Approximately half of the patients also underwent functional occupational therapy and daily living exercises three to five times per week for shoulder dysfunction, and a few patients also underwent physical therapy because whole-body sarcopenia, including physical dysfunction, may affect the swallowing function^18^. Patients were discharged when their surgical wound healed, their complications resolved and they were able to obtain enough nutrients and live at home. If patients still had dysphagia, we provided outpatient rehabilitation or referred them to home-based rehabilitation services.

### Assessment

#### Cross-sectional area of the head and neck muscles

For the assessment of the geniohyoid and masseter muscle masses, two STs were used to measure the preoperative sagittal cross-sectional areas of the geniohyoid muscle^23^ and axial cross-sectional areas of the masseter muscles of the other side of the tumor at 20 slices below the zygomatic arch^21^. The STs were certified under the “Cancer Rehabilitation Educational Program for Rehabilitation Terms” and usually interpreted and analyzed CT examinations for rehabilitation treatment. CT examinations were performed using LightSpeed VCT (General Electric Company, Boston, MA, USA) and Aquilion 16 (Canon Medical Systems Corporation, Tokyo, Japan). The scanning parameters were 120 kVp, 50–325 mAs, and 1-mm slice thickness. Raw data were reconstructed using Aquarius NET software (Tera Recon Inc., Foster City, CA, USA). The mean value for both sides was calculated for each patient. To evaluate the intra-rater and inter-rater reliability of the method, one of the STs assessed the CT data of 12 randomly selected patients from all patients twice, and the other ST, who was blinded to the characteristics of the patients, also assessed the same CT data. We assessed both the intra-rater and inter-rater reliabilities using the intraclass correlation coefficient (ICC).

#### Severity of dysphagia

As the primary outcome, severity of dysphagia was assessed using the Penetration Aspiration Scale (PAS)^27^, which is an 8-point scale that evaluates airway invasion based on the video fluoroscopic swallowing study (VFSS), by two STs after salvage surgery. Some patients underwent VFSS more than once before discharge, and the PAS score at the time of final evaluation before discharge was used in this study. PAS score 1 reflects no entry of material into the airway; PAS scores 2–5 indicates penetration of material; and PAS scores ≥ 6 indicates tracheal aspiration of material. However, because this was a retrospective study, not all patients underwent VFSS.

We also used the Functional Oral Intake Scale (FOIS) score^28^ as the secondary outcome, which ranged from Level 1 (nothing by mouth) to Level 7 (total oral diet with no restrictions). Two STs evaluated the FOIS score, wherein a score below six indicated some limitations in the swallowing function, both at admission and on postoperative day (POD) 90. We also assessed the oropharyngeal swallow efficiency (OPSE), which has been validated for oropharyngeal dysphagia evaluation for patients with head and neck cancer^29^.

### Other parameters

Data on radiotherapy, including “bilateral or unilateral neck irradiation and no neck irradiation”, “radiation dose”, and “combined chemotherapy with radiotherapy (chemoradiotherapy or radiotherapy alone)” before surgery, were used as independent variables. The length of time between radiotherapy and surgery was defined. Surgical lesions, surgical procedures, and whether the geniohyoid muscles were in the radiation fields were also assessed. Data were obtained from surgical and radiation records. The difference between the radiated and non-radiated cross-sectional areas of the masseter muscle was evaluated to assess the effect of radiotherapy on muscle mass in patients who underwent unilateral neck irradiation. Conversely, the geniohyoid muscles on both sides were adjacent to each other and affected by radiotherapy when the submental region was involved in the radiation field in many cases^30^. Thus, we did not measure both sides of the geniohyoid muscle area nor evaluated the difference between the radiated and non-radiated areas of the geniohyoid muscle. It has been reported that low body mass index (BMI) and low Barthel Index (BI) were risk factors for dysphagia^17^. Thus, the BMI was calculated as weight divided by height squared upon admission. Activities of daily living were evaluated using the BI at admission, which was scored on a 0–100-point scale, with higher scores indicating higher levels of independence.

### Statistical analyses

Parametric data were presented as means ± standard deviations, non-parametric data as medians and interquartile ranges (IQRs), and categorical data as percentages. Fisher’s exact test and the Mann–Whitney U test were used to examine sex-dependent differences. Correlations between cross-sectional areas of the head and neck muscles and other parameters were assessed using Spearman’s rank correlation coefficient. The correlation between cross-sectional areas of the geniohyoid muscle and the masseter muscle was evaluated. Multivariate linear regression was used to estimate age-adjusted (Model 1) and age- and surgical lesion and procedure-adjusted (e.g., hypopharynx and larynx with the external approach) (Model 2) standardized partial regression coefficients (β) for the PAS score and OPSE score after surgery and the FOIS score at POD 90. We also performed stratified analysis by age (younger group: under 65 years, and elderly group: 65 years of age and above), site of the surgical lesions (larynx and hypopharynx, oropharynx and others), surgical procedure (with and without reconstruction) and irradiation field used (bilateral or unilateral neck irradiation or no neck irradiation). P-values less than 0.05 were considered statistically significant, and post-hoc power analyses were performed to determine whether the study had an adequate power to detect outcomes. We used the power.t.test function in R to estimate the power in changes in FOIS score. All statistical analyses were performed using EZR (Saitama Medical Center, Jichi Medical University, Saitama, Japan), which is a graphical user interface for R (The R Foundation for Statistical Computing, Vienna, Austria)^31^. More precisely, EZR is a modified version of R commander, designed to add statistical functions frequently used in biostatistics.

This study has been performed in accordance with the ethical standards laid down in the 1964 Declaration of Helsinki and its later amendments and approved by the Ethics Committee of Osaka International Cancer Institute (approval number 1801129313). The Ethics Committee of Osaka International Cancer Institute waived the need for informed consent in view of the retrospective nature of the study. Instead, an opt-out technique through an online announcement on the hospital webpage was used to provide an opportunity for patients to withdraw or to tacitly consent to participate in the study.

### Ethical declarations

The present study was approved by the Ethics Committee of Osaka International Cancer Institute (approval number 1801129313). The Ethics Committee of Osaka International Cancer Institute waived the need for informed consent in view of the retrospective nature of the study. Instead, an opt-out technique through an online announcement on the hospital web page was used to provide an opportunity for patients to withdraw or to tacitly consent to participate in the study.

## Results

### Patient characteristics

Table 1 shows the characteristics of the 45 patients (median age, 68 [IQR 62–72] years) included in this study. Eighteen patients underwent radiotherapy including the geniohyoid muscles.

**Table 1:** Patients’ characteristics.

	All (n = 45)
Age, year (IQR)	68 [62–72]
BMI (IQR)	20.3 [19.0–24.1]
BI (IQR)	100 [100–100]
FOIS score at admission (IQR)	7 [6–7]
**RT with or without chemotherapy (%)**	
RT alone	19 (42.2)
CRT	26 (57.8)
Radiation dose (IQR)	70 [65–70]
**Neck irradiation (%)**	
Unilateral and no neck irradiation	17 (37.8)
Bilateral neck irradiation	28 (62.2)
Irradiation including the GM	16 (35.6)
Time between RT and surgery, month (IQR)	21 [7–68]
FOIS score on POD 90 (IQR)	6 [2–7]
Oropharyngeal swallow efficiency score	95.8 [67.1–116.2]
Penetration Aspiration Scale score	7 [1–8]
CSA of the GM, cm^2^ (SD)	3.13 ± 0.78
CSA of the MM, cm^2^ (SD)	4.70 ± 1.22

*IQR* interquartile range, *BMI* body mass index, *BI* Barthel Index, *FOIS* Functional Oral Intake Scale, *RT* radiotherapy, *CRT* chemoradiotherapy, *CSA* cross-sectional area, *GM* geniohyoid muscle, *MM* masseter muscle, *SD* standard deviation.

Table 2 shows the surgical lesions and surgical procedures. The most common sites were the larynx (31.1%), oropharynx (26.7%), and hypopharynx (24.4%), and 21 patients (46.7%) underwent surgery with free flap reconstruction. The TNM classifications of the recurrent tumor were as follows: rT1N0M0 (n = 19), rT2N0-2M0 (n = 12), rT3N0M0 (n = 1), rT4N0M0 (n = 3), rT0N1-2M0 (n = 6), and necrosis (n = 4).

**Table 2:** Surgical lesions and surgical procedures.

Surgical lesion	Surgical procedure	All (n = 45)
Oropharynx		12 (26.7%)
Tongue base	Partial resection without free flap reconstruction	1
	Partial resection and tonsillectomy with free flap reconstruction	1
Soft palate	Subtotal resection with free flap reconstruction	1
Tonsillar complex	Radical tonsillectomy with free flap reconstruction	6
	Radical tonsillectomy and partial pharyngectomy with free flap reconstruction	3
Hypopharynx		11 (24.4%)
	Partial pharyngectomy with free flap reconstruction	9
	Partial pharyngectomy without free flap reconstruction	1
	Partial pharyngolaryngectomy with free flap reconstruction	1
Larynx		14 (31.1%)
	Vertical partial laryngectomy	9
	Horizontal partial laryngectomy	2
	Wide vertical hemipharyngolaryngectomy	2
	Cricohyoidoepiglottopexy	1
Others		8 (17.8%)
Neck lymph node	Unilateral neck dissection	4
	Bilateral neck dissection	1
Parapharyngeal space	Resection under the transmandibular approach	1
Cervical esophagus	Partial cervical esophagectomy	1
Lateral retropharyngeal node	Retropharyngeal lymph node dissection	1

### Correlation

Spearman’s rank correlation analysis was performed to avoid multicollinearity (Table 3). The BMI was positively correlated with each cross-sectional area of the geniohyoid and masseter muscles and with the implementation of bilateral neck irradiation. The cross-sectional area of the geniohyoid muscle was positively correlated with that of the masseter muscle.

**Table 3:** Spearman’s rank correlation test results.

	CSA of the GM	CSA of the MM	CRT	Bilateral neck irradiation	BMI
Age	− 0.430*	− 0.401*	− 0.08	0.184	− 0.02
BMI	0.531*	0.410*	− 0.218	− 0.522*	–
Bilateral neck irradiation	− 0.220	− 0.222	0.410*	–	
CRT	− 0.147	0.07	–		
CSA of the MM	0.503*	–			

*CSA* cross-sectional area, *MM* masseter muscle, *GM* geniohyoid muscle, *CRT* chemoradiotherapy, *BMI* body mass index.

*P < 0.05.

### Severity of dysphagia and cross-sectional area of the head and neck muscles

For intra-rater reliability, the cross-sectional areas of the geniohyoid and masseter muscles had an ICC of 0.907 and 0.912, respectively; for inter-rater reliability, the cross-sectional areas of the geniohyoid and masseter muscles had an ICC of 0.812 and 0.916, respectively.

The median FOIS score before salvage surgery was 7 (IQR, 6–7). Forty-one patients were able to eat regular food; one patient ate soft bite-sized food; three patients received nasogastric tube feeding without oral intake^28^; no patient had a gastrostomy tube placed prior to or during the salvage surgery. We evaluated the PAS and OPSE scores at median POD 24 (IQR 10–34). The median FOIS score at POD 90 was 6 (IQR 2–7, and the median PAS score and median OPSE score after surgery were 6 (IQR 2–7) and 95.8 (IQR 67.1–116.2), respectively. Eighteen patients had oropharyngeal dysphagia with aspiration after salvage surgery. Sixteen patients who were expected to have insufficient total energy intake for longer than 60 days after surgery had a gastrostomy tube placed or received intermittent oral esophageal tube feeding after postoperative VFSS. The mean geniohyoid muscle cross-sectional area was 3.13 ± 0.78 cm^2^; the mean masseter muscle cross-sectional area was 4.37 ± 0.99 cm^2^. Patients, who underwent unilateral neck irradiation, showed no significant differences between the radiated and non-radiated cross-sectional areas of the masseter muscle.

Table 4 shows the age-adjusted (Model 1) and age-,and surgical lesion and procedure-adjusted (Model 2) β coefficients for the PAS and OPSE scores after surgery and the FOIS score at POD 90. Model 1 shows that the cross-sectional area of the geniohyoid muscle was significantly associated with the PAS score after surgery (β =  − 2.15, 95% confidence interval [CI] − 3.31 to (− 0.98), β =  − 0.35); however, that of the masseter muscle was not associated with the PAS score after surgery (β =  − 0.37, 95% CI − 1.24 to 0.49, β =  − 0.14). The cross-sectional area of the geniohyoid muscle (β = 1.6, 95% CI 0.68–2.52, β = 0.42) and of the masseter muscle (β = 0.64, 95% CI 0.10–1.18, β = 0.30) were both significantly associated with the FOIS score at POD 90. The post-hoc power of the study was 0.99. The preoperative FOIS score was not significantly associated with the PAS score after surgery, FOIS score at POD 90, and OPSE score. Preoperative cross-sectional areas of the geniohyoid and masseter muscles were not associated with the length of hospitalization. Tables 5 and 6 show the stratified analysis. In the elderly group, preoperative geniohyoid muscles were significantly associated with the PAS, FOIS, and OPSE scores.

**Table 4:** Multivariate analysis for the Penetration Aspiration Scale score, FOIS score, and oropharyngeal swallow efficiency score after surgery.

	PAS				FOIS				OPSE			
	Beta	95% CI	β	P-value	Beta	95% CI	β	P-value	Beta	95% CI	β	P-value
**CSA of GM**												
Model 1												
Age	− 0.1	− 0.21 to − 0.015	− 0.16	0.08	0.01	− 0.07 to 0.11	− 0.02	0.762	0.21	− 1.34 to 1.77	− 0.03	0.783
CSA of GM	− 2.15	− 3.31 to − 0.98	− 0.35	< 0.001	1.6	0.68–2.52	0.42	0.001	30.93	15.4–46.4	0.39	< 0.001
Model 2												
Age	− 0.1	− 0.22 to 0.01	− 0.16	0.089	0.01	− 0.07 to 0.11	− 0.02	0.751	0.27	− 1.37 to 1.79	− 0.03	0.786
Surgical procedure	− 0.27	− 2.02 to 1.46	− 0.01	0.747	0.82	− 0.52 to 2.17	0.17	0.223	0.77	− 22.37 to 23.92	− 0.03	0.946
CSA of GM	− 2.12	− 3.31 to − 0.93	− 0.35	< 0.001	1.53	0.61–2.45	0.39	0.002	30.86	15.02–46.71	0.38	< 0.001
**CSA of MM**												
Model 1												
Age	− 0.04	− 0.17 to 0.09	− 0.11	0.517	− 0.01	− 0.16 to 0.05	− 0.05	0.828	− 0.14	− 1.14 to 0.84	− 001	0.764
CSA of MM	− 0.37	− 1.24 to 0.49	− 0.14	0.391	0.64	0.10–1.18	0.3	0.02	15.79	6.68–24.89	0.45	0.001
Model 2												
Age	− 0.04	− 0.17 to 0.09	− 0,11	0.522	− 0.004	− 0.11 to 0.08	− 0.05	0.731	− 0.03	− 1.69 to 1.623	− 0.01	0.967
Surgical procedure	− 0.21	− 2.28 to 1.84	− 0.04	0.828	0.78	− 0.71to 2.27	0.17	0.296	− 4.33	− 29.8 to 21.1	− 0.05	0.732
CSA of MM	− 0.33	− 1.27 to 0.59	− 0.13	0.466	0.48	− 0.19 to 1.16	0.24	0.154	16.91	5.37–28.4	0.47	0.005

Model 1: Age-adjusted.

Model 2: Age-, and surgical lesion and procedure-adjusted.

*PAS* penetration aspiration scale, *FOIS* Functional Oral Intake Scale, *OPSE* oropharyngeal swallow efficiency, *CSA* cross-sectional area, *GM* geniohyoid muscle, *MM* masseter muscle, *CI* confidence interval.

**Table 5:** Stratified analyses between the cross-sectional area of the geniohyoid muscle and PAS, FOIS and OPSE scores after surgery.

	N	PAS			FOIS			OPSE		
		Beta	95% CI	P-value	Beta	95% CI	P-value	Beta	95% CI	P-value
**Age**										
< 65 years	19	− 0.55	− 2.43 to 1.32	0.541	0.83	− 0.71 to 2.371	0.268	11.16	− 13.81 to 36.12	0.357
≧ 65 years	26	− 1.85	− 3.58 to − 0.14	0.035	1.68	0.56–2.81	0.005	30.43	7.68–53.19	0.011
**Surgical lesions**										
Larynx and hypopharynx	24	− 1.04	− 2.90 to 0.82	0.256	1.05	− 0.22 to 2.33	0.101	21.163	− 5.629 to 47.957	0.114
Oropharynx	13	− 1.91	− 4.19 to 0.38	0.091	1.92	− 0.21 to 3.63	0.031	33.19	3.89–62.49	0.029
Others	8	− 1.20	− 3.48 to 1.06	0.231	1.60	− 0.15 to 3.36	0.064	14.44	− 23.21 to 52.10	0.369
**Surgical procedures**										
With reconstruction	23	− 0.77	− 2.42 to 0.86	0.335	0.97	− 0.48 to 2.43	0.177	24.82	− 0.30 to 49.94	0.052
Without reconstruction	22	− 1.35	− 3.10 to 0.40	0.123	1.34	0.22–2.46	0.021	15.98	− 3.45 to 35.14	0.101
**Irradiation field**										
Bilateral neck irradiation	28	− 0.99	− 2.47 to 0.49	0.179	1.39	0.344–2.44	0.011	16.03	− 4.23 to 36.27	0.115
Unilateral or no neck irradiation	17	− 1.19	− 3.61 to 1.21	0.304	0.247	− 1.12 to 1.62	0.705	19.83	− 7.63 to 47.03	0.140

*N* number of patients, *PAS* penetration aspiration scale, *FOIS* Functional Oral Intake Scale, *OPSE* oropharyngeal swallow efficiency, *CI* confidence interval.

**Table 6:** Stratified analyses between the cross sectional area of the masseter muscle and the PAS, FOIS, and OPSE scores after surgery.

	N	PAS			FOIS			OPSE		
		Beta	95% CI	P-value	Beta	95% CI	P-value	Beta	95% CI	P-value
**Age**										
< 65 years	19	− 0.03	− 1.63 to 1.68	0.967	0.34	− 1.03 to 1.72	0.599	15.08	− 5.76 to 35.91	0.144
≧ 65 years	26	− 0.38	− 1.37 to 0.64	0.458	0.55	− 0.12 to 1.23	0.104	15.25	2.79–27.71	0.019
**Surgical lesions**										
Larynx and hypopharynx	24	− 0.35	− 1.71 to 1.00	0.592	0.42	− 0.52 to 1.38	0.361	16.47	− 2.29 to 35.24	0.081
Oropharynx	13	− 0.29	− 1.61 to 1.02	0.634	0.68	− 0.31 to 1.69	0.157	21.61	9.67–33.56	0.002
Others	8	− 0.28	− 2.41 to 1.85	0.748	0.46	− 1.51 to 2.45	0.57	23.42	3.82–43.02	0.027
**Surgical procedures**										
With reconstruction	23	0.01	− 1.02 to 10.58	0.971	0.75	− 0.14 to 1.65	0.097	10.25	− 6.31 to 26.82	0.210
Without reconstruction	22	− 0.29	− 1.51 to 0.91	0.612	0.26	− 0.56 to 1.09	0.516	15.73	4.41–27.05	0.009
**Irradiation field**										
Bilateral neck irradiation	28	− 0.00	− 0.09 to 0.98	0.998	0.53	− 0.19 to 1.26	0.145	13.05	0.62–25.48	0.04
Unilateral or no neck irradiation	17	− 0.47	− 1.99 to 1.03	0.509	0.00	− 0.83 to 0.85	0.980	12.36	− 4.33 to 29.06	0.134

*N* number of patients, *PAS* penetration aspiration scale, *FOIS* Functional Oral Intake Scale, *OPSE* oropharyngeal swallow efficiency, *CI* confidence interval.

## Discussion

This retrospective study involving 45 male patients who underwent salvage surgery after radiotherapy for head and neck cancer, examined the association between the severity of dysphagia and both geniohyoid and masseter muscle masses as evaluated using neck CT. The main finding of this study was that the cross-sectional areas of the geniohyoid muscle evaluated using preoperative neck CT were associated with the severity of dysphagia after salvage surgery.

The preoperative geniohyoid muscle mass was associated with the severity of dysphagia evaluated using both the PAS and FOIS scores after salvage surgery. It is essential for patients undergoing unilateral or bilateral resection of the suprahyoid muscles after surgery for head and neck cancer that the remaining suprahyoid muscles maintain their swallowing function. Otherwise, the swallowing function may decline in patients with reduced suprahyoid muscle mass. Recently, some studies have reported that reduced whole-body skeletal muscle mass and swallowing muscle mass, as well as degradation of the whole-body muscles and swallowing muscles, cause sarcopenic dysphagia^16–19,32^. In the present study, we did not assess whole-body skeletal muscle mass; however, geniohyoid muscle mass was associated with the severity of dysphagia after surgery and may show an association with sarcopenic dysphagia. Feng et al. assessed the cross-sectional area of the geniohyoid muscle on CT and found that it was significantly smaller in aspirators than in non-aspirators^24^. In addition, an association between dysphagia and decreased muscle mass in the muscles related to the swallowing function, such as the digastric, temporal, geniohyoid, and pharyngeal muscles, has been reported^23,33^. Their results suggest that the geniohyoid muscle mass was associated with the swallowing function, consistent with our study. The OPSE score may be an indicator of the mechanism of dysphagia; it measures patients’ ability to transport bolus to the esophagus. The presence of pharyngeal residue as well as aspiration affects the OPSE score. In the present study, the geniohyoid muscle mass was associated with both PAS and OPSE scores, suggesting that the lack of laryngeal movement causes pharyngeal residue as well as aspiration. As the geniohyoid muscle affects oropharyngeal swallowing, patients with lower geniohyoid muscle mass may develop subclinical dysphagia. Moreover, the stratified analysis suggests that the cross-sectional area of the geniohyoid muscle was associated with swallowing function after salvage surgery in the elderly group, although there were no significant associations in the younger group. Thus, patients with decreased geniohyoid muscle mass may be affected by sarcopenia as well as salvage surgery, leading to more severe dysphagia. Therefore, we believe that preoperative geniohyoid muscle mass may predict severity of dysphagia after salvage surgery. We suggest several options for patients with decreased preoperative geniohyoid muscle mass, such as gastrostomy tube placement prior to or during salvage surgery, preoperative nutritional management and rehabilitation treatment of the swallowing muscle, or alteration of the surgical procedure.

The hypothesis that the masseter muscle mass evaluated with preoperative neck CT would predict severity of dysphagia according to PAS score in patients undergoing salvage surgery and radiotherapy for head and neck cancer was not confirmed in the present study. The masseter muscle is one of the swallowing muscles but not part of the suprahyoid muscles. Thus, we consider that the weakness of masseter muscle affects eating ability rather than swallowing function. In the present study, the relationship between the masseter muscle mass and FOIS score at POD 90 and OPSE score predicts that the masseter muscle mass affects the pharyngeal and oral phases of swallowing. A previous study has reported that masseter muscle mass was associated with whole-body muscle mass^21^. Thus, whole-body sarcopenia may be associated with the OPSE score. Moreover, the masseter muscle mass affects the oral phase of swallowing, e.g. mastication, because the FOIS score focuses on food texture as well as the feeding route^28^ and may represent the oral condition. There are reports that the masseter muscle mass is associated with oral abilities, such as masticatory performance and maximal bite force^34,35^. Mastication is important for the formation of a coherent bolus safe enough for swallowing^35^. Thus, in the current study, the swallowing function evaluated using the FOIS score may have been affected by the mastication ability and masseter muscle mass.

Skeletal muscle mass is often evaluated in patients with cancer using abdominal CT to examine the psoas muscle; however, abdominal CT is not performed routinely in patients with head and neck cancer. In contrast, evaluation of geniohyoid and masseter muscle mass using neck CT is easy and effective for most patients with head and neck cancer. We posit that evaluation of geniohyoid or masseter muscle mass with neck CT may be useful for the diagnosis of sarcopenia in patients with head and neck cancer.

Both geniohyoid and masseter muscle masses were lower in the present study than in previous studies that examined older blunt trauma cases and healthy elderly individuals^21,23^. Radiotherapy for head and neck cancer induces atrophy of the muscle caused due to fibrosis, nerve degeneration, and damage to the small vessels^36^. Moreover, it may also be affected by disuse atrophy of those muscles owing to the side effects of chemoradiotherapy or radiotherapy, including poor oral intake or need for a dysphagia diet. In addition, malnutrition is common in patients with cancer owing to symptoms of the disease and side effects of treatments, such as radiotherapy, chemotherapy, and surgery^7,8^. Unsal et al. reported a malnutrition rate of 88% in patients with head and neck cancer after radiotherapy^9^. Malnutrition causes decreased skeletal muscle mass and sarcopenia, which are risk factors for dysphagia^16,18,19^. Because the combination of both rehabilitation and nutritional management is indispensable to increase muscle mass and to treat sarcopenic dysphagia^37^, appropriate rehabilitation and nutritional treatment before HNC treatment may increase the swallowing muscle mass and improve dysphagia.

There are several limitations in this study. First, we could not adjust for many factors, such as radiation dosage, time of assessment of the PAS score, radiation site, irradiation range, surgical procedure, nutritional status, muscle strength and length of hospitalization, as they varied widely. Second, although our institute is among the largest cancer centers treating HNC, our sample size was small because this study included only male patients who had recurrence or necrosis after treating HNC and we excluded patients who had undergone resection of the geniohyoid muscle or ansa cervicalis, female patients, and patients with oral cancer to lessen the impact of surgery for swallowing function. Thus, our findings may not be generalized to all HNC patients. However, the age- and surgical procedure adjusted β coefficients were calculated to verify whether preoperative geniohyoid and masseter muscle mass can predict dysphagia after surgery, and the statistical power was acceptable. Third, fibrosis after radiotherapy was not assessed, although radiotherapy causes secondary fibrosis of the pharyngeal muscles and soft tissues, leading to muscle weakness and oropharyngeal dysphagia^2^. However, several studies have reported skeletal muscle atrophy and fibrosis after irradiation therapy^38^. Therefore, radiation-induced fibrosis may have resulted in muscle mass atrophy or muscle mass loss in the present study. Fourth, we did not evaluate VFSS for all patients before salvage surgery, therefore it is conceivable that there were patients with only mild subclinical dysphagia before salvage surgery, because the preoperative median FOIS score was 7 (IQR 7–7).

In conclusion, the preoperative geniohyoid muscle mass evaluated using neck CT is a potential predictor for dysphagia after salvage surgery. Further research is required to determine whether preoperative treatment, including nutrition support and exercise of the head and neck muscles, will prevent occurrence of severe dysphagia.
